# Enhancing patient engagement through an online educational module for gender-affirming mastectomy: A pilot study^☆^

**DOI:** 10.1016/j.jpra.2026.05.003

**Published:** 2026-05-15

**Authors:** Nina R. Mehta, Matthew Mclaughlin, Israel Falade, Siyou Song, Chelsea Yim, Esther A. Kim

**Affiliations:** a University of North Carolina at Chapel Hill School of Medicine, USA; b University of California San Francisco School of Medicine, San Francisco, CA, USA; c University of California San Francisco Division of Plastic & Reconstructive Surgery, San Francisco, CA, USA

**Keywords:** Gender-affirming surgery, Patient education, Top surgery, Gender dysphoria, Shared decision-making, Pilot study

## Abstract

**Background and Purpose:**

Patients seeking gender-affirming mastectomy (GAM) frequently encounter barriers to care, including limited access to accurate and comprehensive preoperative education regarding surgical options. We conducted a pilot study to evaluate the effectiveness of an online educational module in improving patient understanding of GAM, reducing procedure-related anxiety, and increasing satisfaction with the surgical consultation.

**Methods:**

We developed a standardized online educational module delivering expert-reviewed information on GAM. Patients presenting for initial GAM consultation were randomly assigned using a computer-generated sequence to either a control arm (consultation only) or an intervention arm (consultation plus educational module). The consulting surgeon was blinded to group allocation. Pre- and post-consultation surveys assessed surgical readiness, encompassing procedural knowledge and anxiety, using a 7-point Likert scale. Post-consultation satisfaction was also evaluated. Statistical analyses included the Mann-Whitney U Test, chi-squared or Fisher’s exact tests, and two-way repeated measures ANOVA with Bonferroni-corrected post hoc analyses.

**Results:**

Thirty-six patients were enrolled (18 intervention; 18 control). The intervention group had significantly higher post-consultation surgical readiness scores compared with their own pre-consultation scores (p=0.0002) and with post-consultation scores in the control group (p=0.0032). Post-consultation satisfaction scores were higher in the intervention arm, although this difference did not reach statistical significance (p=0.0579). All patients in the control group indicated that an additional educational resource would have been helpful.

**Conclusion:**

In this pilot study, an online educational module for GAM significantly improved patient-reported surgical readiness and reduced perioperative anxiety. This approach enhances patient preparedness and promotes shared surgical decision-making. Larger studies are needed to confirm these findings and assess the effect of preoperative education on perioperative outcomes.

## Introduction

The number of individuals in the United States identifying as LGBTQ+ has increased steadily in recent years.^1^ According to Gallup, 7.1% of Americans identify as LGBTQ+, with approximately 0.7% identifying as transgender.^1^ This demographic shift has been accompanied by an increasing demand for gender-affirming surgeries (GAS), including gender-affirming mastectomy (GAM). GAM has been shown to align physical appearance with gender identity and to significantly improve anxiety, depression, and overall psychosocial functioning.^2^ Despite the increasing rate of GAS, many patients face barriers within healthcare systems, including limited access to accurate, comprehensive, and affirming educational resources.

Meaningful patient involvement in preoperative decision-making is essential for achieving surgical outcomes that reflect individual goals and values. This process depends on a clear understanding of all surgical options, risks, and expectations, traditionally facilitated during physician consultations. However, time constraints and structural limitations in clinical practice often restrict the depth of education that can be provided. Consequently, patients may turn to internet searches, online forums, or social media, where information is frequently inconsistent, biased, or inaccurate, potentially influencing surgical expectations and decisions.^3^

To enhance the surgical consultation experience and improve patient understanding, we created a standardized online educational module focused on GAM. The module was designed to provide expert-reviewed, high-quality information regarding surgical techniques, perioperative expectations, and postoperative care. By equipping patients with foundational knowledge before consultation, the module aimed to allow surgeons to focus on individualized goals, values, and shared decision-making. This pilot study evaluated the effectiveness of the educational module in improving procedural understanding, reducing anxiety, and increasing satisfaction with the surgical consultation. We hypothesized that patients who reviewed the module would demonstrate greater knowledge, reduced anxiety, and higher satisfaction following consultation.

## Methods

### Study design

We conducted a prospective, single-blinded pilot study from May 2023 to October 2023. This study was approved by the Institutional Review Board (22-35978), and informed consent was obtained from all participants. The study was designed as a pilot investigation to assess the feasibility and preliminary efficacy of a preoperative online educational module for GAM. A formal a priori power analysis was not performed given the exploratory nature of this investigation, which represents the first prospective evaluation of an educational intervention specific to GAM. The primary objective was to improve patient understanding of GAM and support informed decision-making.

### Enrollment and randomization

Eligible participants were English-speaking individuals aged 18 or older presenting for initial GAM consultation who met the World Professional Association for Transgender Health (WPATH) criteria. A total of 43 patients were recruited; seven were excluded due to incomplete survey completion, resulting in a final cohort of 36 participants. Patients were randomly assigned to either a control arm (consultation only) or an intervention arm (consultation plus educational module) using a computer-generated random number sequence with a 1:1 allocation ratio. The allocation sequence was maintained in a secure electronic spreadsheet accessible only to the study coordinator, and participants were sequentially assigned upon enrollment. Participants in the intervention arm received a secure email link to the module five days prior to consultation. The consulting surgeon remained blinded to group assignment throughout the study. Participants could not be blinded to their group allocation due to the nature of the educational intervention.

### Online educational module

The educational intervention consisted of an 8-minute narrated video module. The module was created by the first author and reviewed for clinical accuracy by the senior author, a board-certified plastic surgeon specializing in gender-affirming surgery. The first author recorded a voiceover narration based on a script that was reviewed and approved by the senior author. The module was organized into five content sections: (1) surgical candidacy criteria, (2) procedural techniques with visual illustrations of incision patterns and expected outcomes, (3) perioperative timelines, (4) postoperative care instructions, and (5) potential risks and complications. The module concluded with contact information for follow-up questions or further inquiries.

Content was written in accessible, layman’s terms to maximize comprehension across varying levels of health literacy. Of note, the module was not subjected to formal psychometric validation or readability testing, and we therefore describe it as clinically reviewed rather than validated. The slide content of the module is provided in Supplemental Digital Content 4; patient photographs have been removed to protect privacy, and the full narrated module is available from the corresponding author upon reasonable request.

### Pre- and post-consultation surveys

Three surveys were developed and administered electronically to both study arms before and after consultation. Survey instruments were adapted from Dayucos et al.^4^ and modeled on the validated Perceived Health Website Usability Questionnaire (PHWSUQ).^5^ Surveys assessed surgical readiness, defined as a composite construct encompassing procedural knowledge and anxiety, and post-consultation satisfaction.

Responses were recorded on a 7-point Likert scale (1 = strongly disagree; 7 = strongly agree). The surgical readiness composite score comprised 11 items (maximum 77 points). Items 2 through 4 and 6 through 9 assessed procedural knowledge and perioperative preparedness (e.g., understanding of surgical techniques, risks, hospital arrival expectations, and recovery steps). Items 10 through 12 assessed psychosocial and financial readiness, including perceived social support for preparation and recovery and financial preparedness. These items were included because psychosocial and practical readiness are recognized components of overall surgical preparedness in the shared decision-making literature, particularly in gender-affirming surgery where support systems and financial coverage can substantially influence decision-making and outcomes. Item 5 (“I am anxious about my upcoming procedure”) was reverse-scored such that higher scores reflected lower anxiety. In the composite score, higher values therefore consistently indicate greater readiness (i.e., greater knowledge, more perceived support, and less anxiety). Full survey instruments are provided in the Supplemental Digital Content.

### Statistical analysis

Descriptive statistics were calculated for demographic variables. Continuous demographic variables were summarized as medians and interquartile ranges (IQR) and compared between arms using the Mann-Whitney U Test. Categorical variables were described by frequency and percentages and compared using chi-squared or Fisher’s exact tests.

Surgical readiness scores were analyzed as means ± standard error (SE), consistent with the parametric ANOVA framework**.** Two-way repeated measures ANOVA investigated intervention group and time effects on surgical readiness scores. The intervention group served as a between-subjects factor with two levels (i.e., educational module plus consultation vs. consultation only), while time was a within-subjects factor with two levels indicating the time points at which measurements were taken (i.e., pre- and post-consultation). The significance level was set at alpha = 0.05. When significant main effects or interactions were detected, post hoc analyses with Bonferroni correction were performed to explore the differences between specific means. Patients in both arms answered 7 questions regarding satisfaction with the consultation, assessed on a 7-point Likert scale. The total possible score for these measures was 49 points. An independent t-test examined post-consultation satisfaction scores between the intervention arm (consultation plus educational module) and the control arm. Finally, post-consultation satisfaction questions that were unique to each arm are presented as the percentages of respondents answering each Likert scale option. All analyses were conducted in Stata 17.0 (StataCorp, College Station, Texas).

## Results

### Patient demographics

The median age of all 36 patients in the study was 29 (IQR: 25–39). Most participants identified as White or Caucasian (58.3%) and were employed (69.4%). All participants had at least a high school education, and 94.4% reported no prior GAS. Mental health comorbidities were reported by 38.9% of participants, most commonly depression (27.8%). Participants in the control arm were significantly older than those in the intervention arm (median 36 vs. 26.5 years; p=0.016);); no other demographic differences were observed (Table 1).

**Table 1:** Patient Demographics.

Characteristic	Total cohort (n = 36)	Educational Module + Consult (n = 18)	Consult only (n = 18)	P value
Age, yr				
Median	29	26.5	36	0.016*
IQR	25–39	22–29	28–43	
Race/ethnicity				
White/Caucasian	21 (58.3)	10 (55.6)	11 (61.1)	0.477
Hispanic/Latino	4 (11.1)	2 (11.1)	2 (11.1)	
Asian or Pacific Islander	4 (11.1)	3 (16.7)	1 (5.6)	
Black or African American	3 (8.3)	0 (0.0)	3 (16.7)	
Native American or Alaskan				
Native	1 (2.9)	1 (5.6)	0 (0.0)	
Mixed	3 (8.3)	2 (11.1)	1 (5.6)	
Education				
Higher secondary school	12 (33.3)	5 (27.8)	7 (38.9)	0.303
High school	8 (22.2)	6 (33.3)	2 (11.1)	
Graduate or postgraduate	16 (44.4)	7 (38.9)	9 (50.0)	
Employment status				
Employed	25 (69.4)	10 (55.6)	15 (83.3)	0.089
Student	7 (19.4)	4 (22.2)	3 (16.7)	
Unemployed	4 (11.1)	4 (22.2)	0 (0.0)	
Any mental health comorbidity	14 (38.9)	5 (27.8)	9 (50.0)	0.171
Specific mental health comorbidities				
Depression	10 (27.8)	4 (22.2)	6 (33.3)	
Anxiety	5 (13.9)	3 (16.7)	2 (11.1)	
Bipolar disorder	1 (2.8)	1 (5.6)	0 (0.0)	
PTSD	1 (2.8)	1 (5.6)	0 (0.0)	
Prior gender-affirming surgery	2 (5.6)	2 (11.1)	0 (0.0)	0.486

### Knowledge and anxiety scores

**Fig. 1:**
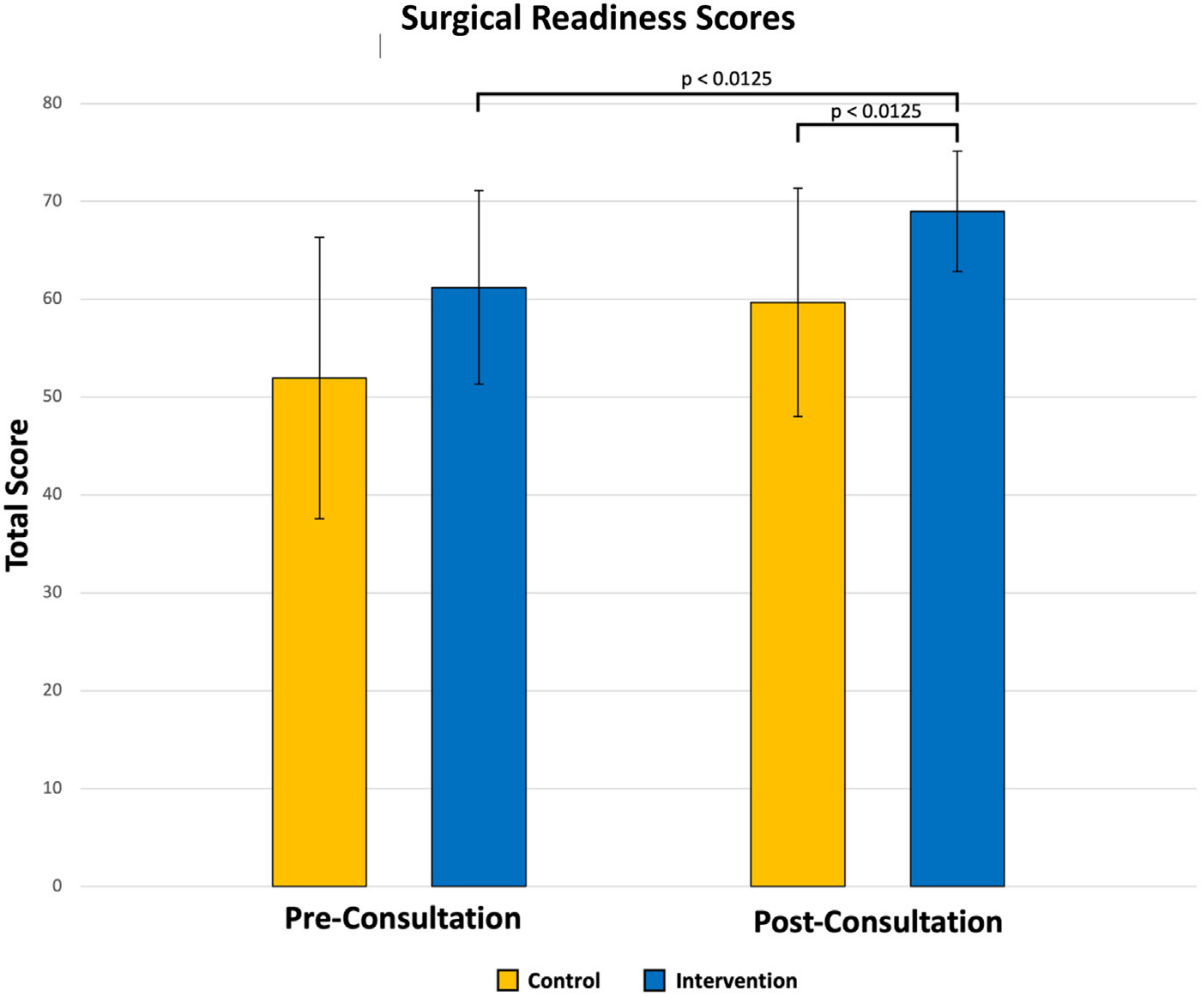
Mean (±SE) pre- and post-consultation surgical readiness scores by study arm. Significant differences after the Bonferroni correction are indicated (i.e., p < 0.0125).

All participants in the intervention arm confirmed that they had reviewed the educational module. Mean (±SE) pre- and post-consultation surgical readiness scores are depicted in Fig. 1. Two-way repeated measures ANOVA revealed significant main effects for intervention group (F(1, 34)=8.50, p=0.0062) and time (F(1, 34)=22.92, p<0.0001), indicating independent effects on surgical readiness scores. The interaction between group and time was not significant (F(1, 34)=0.02, p=0.8943), indicating that the magnitude of improvement from pre- to post-consultation was similar in both groups. However, the significant main effect of group demonstrates that intervention participants had consistently higher surgical readiness scores across both time points, reflecting an additive benefit of the educational module on top of the consultation itself.

Post hoc analyses revealed a significant increase in surgical readiness scores among the intervention participants from pre- to post-consultation (mean 69.4±1.2 vs. 61.2±2.3, p=0.0002). At the post-consultation time point, the intervention participants also demonstrated significantly higher readiness scores compared with control participants (mean 69.4±1.2 vs. 59.8±2.8, p=0.0032) (Fig. 1).

### Post-consultation satisfaction scores

**Fig. 2:**
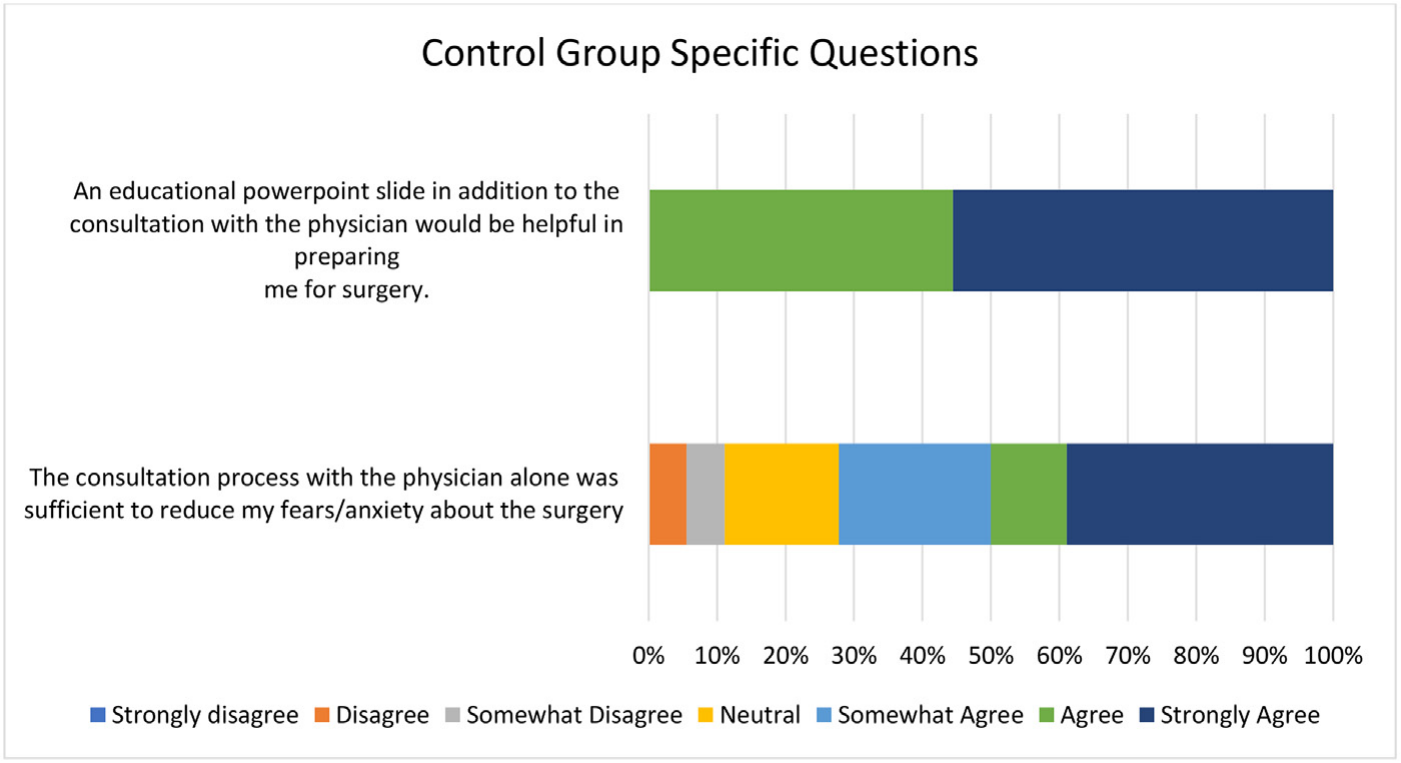
Post-consultation control group-specific questions regarding patient satisfaction (n = 18 respondents).

**Fig. 3:**
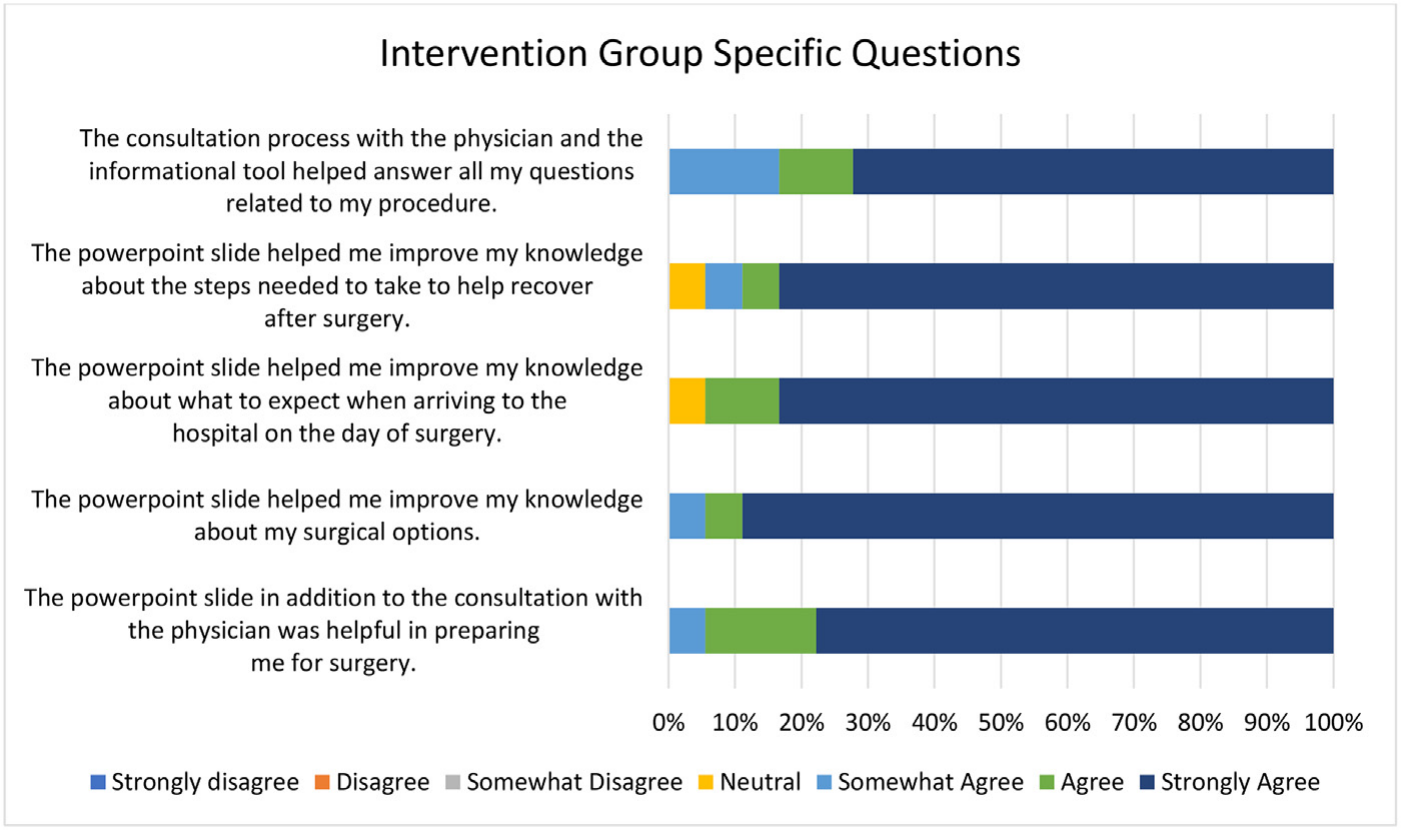
Post-consultation intervention group-specific questions regarding patient satisfaction (n = 18 respondents).

Post-consultation satisfaction scores tended to be higher among patients in the intervention arm than among those in the control arm, although the difference did not reach statistical significance (mean 37.8±1.3 vs. 33.6±1.7, p=0.0579). All control participants agreed or strongly agreed that an additional educational resource would have been beneficial (Fig. 2). Participants in the intervention arm reported high satisfaction with the educational module, with all indicating that it helped address their questions when used in conjunction with consultation (Fig. 3).

## Discussion

This pilot study represents the first prospective evaluation of a standardized online educational module designed specifically for patients undergoing GAM consultation. Our findings demonstrate that providing structured, clinically reviewed educational content before surgical consultation significantly improves patient-reported surgical readiness and reduces procedure-related anxiety. These results underscore the value of supplementing traditional consultation models with asynchronous, patient-centered educational tools, particularly in the context of gender-affirming surgery, where misinformation and variability in counseling remain common.

Consistent with our hypothesis, patients who reviewed the educational module demonstrated significantly higher surgical readiness scores compared with both their own pre-consultation scores and post-consultation scores of patients who underwent consultation alone. Surgical readiness, as defined by a composite of procedural knowledge and reduced anxiety, is a critical component of high-quality surgical decision-making. By improving baseline understanding before consultation, the educational module appears to prime patients for more effective engagement, allowing consultation time to be used for clarification, values-based discussion, and individualized surgical planning rather than foundational education.

The ANOVA results provide additional nuance to the intervention effect. The non-significant interaction between group and time indicates that both groups improved at a similar rate from pre- to post-consultation, suggesting that the consultation itself conferred benefit regardless of group assignment. However, the significant main effect of group indicates that the intervention group had higher readiness scores at both time points. This pattern suggests that the educational module provided an additive benefit: patients who viewed the module entered the consultation with a higher baseline level of understanding, and this advantage was maintained after the consultation. The intervention effect therefore reflects the module’s ability to elevate the starting point for the surgical conversation rather than altering its trajectory.

Our findings align with a growing body of literature supporting the role of preoperative education in improving patient-reported outcomes across surgical disciplines. Prior studies have shown that structured educational interventions reduce perioperative anxiety, improve procedural comprehension, and enhance patient confidence for surgeries ranging from orthopedic arthroplasty to thoracic and image-guided procedures.^4^, ^5^, ^6^, ^7^ Jamwal et al. demonstrated reduced anxiety and depression following a structured perioperative education program for chest surgery patients, while Dayucos et Al. reported improved knowledge among arthroplasty patients using a web-based educational platform.^4^^,^^6^ Our study extends these findings to the field of gender affirming surgery, an area in which patients often rely heavily on non-peer-reviewed online sources due to limited access to standardized educational materials.

Importantly, gender-affirming surgery presents unique educational challenges. Patients often arrive at consultation with highly variable baseline knowledge, shaped by online forums, social media, and anecdotal experiences. While peer-to-peer information sharing can be affirming, it may also propagate misinformation regarding surgical candidacy, technique selection, complication rates, and recovery expectations. A clinically reviewed, institutionally developed educational module helps mitigate these risks by providing consistent, evidence-based information while still allowing surgeons to tailor recommendations to individual patient goals.

Although post-consultation satisfaction scores did not differ significantly between groups, the observed trend toward higher satisfaction in the intervention arm suggests a potential benefit that may not have been fully captured due to the limited sample size. Satisfaction is a multifactorial construct influenced by interpersonal communication, institutional experience, and individual expectations. It is plausible that improved baseline understanding enhances satisfaction directly by fostering more meaningful dialogue, aligning expectations, and increasing patient confidence in the surgical plan. Future studies incorporating postoperative satisfaction measures or longer-term follow-up may better elucidate the relationship between preoperative education and patient satisfaction.

Beyond patient experience, reducing perioperative anxiety may have important clinical implications. Elevated anxiety and psychological distress have been associated with increased anesthetic requirements, prolonged hospital stays, impaired wound healing, higher infection rates, and increased readmission rates,^8^, ^9^, ^10^, ^11^ While this pilot study measured patient-reported readiness and anxiety rather than clinical outcomes directly, the observed improvements in these measures suggest that educational interventions such as the one evaluated here may contribute to downstream improvements in perioperative outcomes and resource utilization. Future studies with larger cohorts should evaluate whether these patient-reported benefits translate into measurable clinical and economic outcomes.

An important consideration in interpreting these results is the significant age difference between the study arms (median 26.5 years in the intervention group vs. 36 years in the control group). Younger patients may have greater digital literacy and comfort with online educational platforms, which could have influenced engagement with the module or baseline readiness scores. Additionally, younger patients may have different patterns of preoperative information-seeking behavior, including greater use of social media and online communities. The small sample size precluded age-adjusted analysis, and we cannot rule out that age differences between groups may have influenced the results. Future studies with larger samples should account for age in their analyses to better isolate the effect of the educational intervention.

This pilot study has several important limitations. First, the sample size of 36 participants (18 per arm) was modest and limits statistical power, the ability to detect smaller effect sizes, and the generalizability of our findings. A formal a priori power analysis was not performed; however, the effect sizes observed in this study can inform sample size calculations for future confirmatory trials. Second, the study population was predominantly White and Asian, English-speaking, and highly educated, recruited from a single academic medical center, which may not reflect the broader and more diverse population seeking gender-affirming care. Third, participants could not be blinded to their group assignment, which may have introduced expectation bias or a Hawthorne effect among those who received the module. Fourth, the survey instrument, although adapted from previously published tools and modeled on a validated questionnaire, was not independently validated for this specific population; future studies should consider formal psychometric evaluation. Fifth, mental health comorbidities, which are prevalent among transgender and gender-diverse individuals, may influence anxiety, comprehension, and decision-making, and were not specifically stratified in outcome analyses. Despite these limitations, the study’s prospective design and blinding of the consulting surgeon strengthen the validity of the findings. The universal endorsement of an educational tool by control participants further highlights an unmet need within standard consultation workflows.

Future research should focus on expanding this approach to other gender-affirming procedures, adapting modules for diverse linguistic and cultural populations, and exploring personalized educational strategies that take into account mental health status, learning preferences, and access barriers. Item-level analysis of individual survey responses would help identify which specific domains of surgical readiness are most responsive to educational intervention and inform the development of more targeted modules. Larger, multicenter studies are needed to confirm these preliminary findings and to assess the effect of preoperative education on clinical, psychosocial, and economic outcomes.

Incorporating standardized educational tools in gender-affirming surgical care represents a scalable and patient-centered strategy to improve preparedness, support shared decision-making, and promote equitable access to high-quality information. As demand for gender-affirming surgery continues to rise, such interventions may play an increasingly important role in optimizing both patient experience and clinical care.

## Conclusion

In this pilot study, implementation of a standardized online educational module for GAM significantly improved patient-reported surgical readiness and reduced perioperative anxiety. This approach enhances patient preparedness and supports shared decision-making. These preliminary findings warrant confirmation in larger, multicenter studies and represent a scalable strategy to improve the quality of preoperative care in gender-affirming surgery.

## Funding

None.

## Conflicts of interest

None declared.

## Ethical approval

This study was approved by the Institutional Review Board (IRB #22-35978). Informed consent was obtained from all participants.
